# Ghrelin produces antidepressant-like effect in the estrogen deficient mice

**DOI:** 10.18632/oncotarget.19768

**Published:** 2017-08-01

**Authors:** Jie Fan, Bing Jin Li, Xue Feng Wang, Li Li Zhong, Ran Ji Cui

**Affiliations:** ^1^ Jilin Provincial Key Laboratory on Molecular and Chemical Genetics, The Second Hospital of Jilin University, Changchun, Jilin 130041, P.R. China

**Keywords:** ghrelin, depression, antidepressant, estrogen, ovariectomize

## Abstract

Recent evidence shows that ghrelin plays an important role in depression. However, it was little known whether ghrelin produces antidepressant-like effect in the ovariectomized mice. The present study was aimed to investigate the antidepressant-like effects of the ghrelin in ovariectomized mice. In the forced swim test, ghrelin significantly decreased immobility time, reversing the “depressive-like” effect observed in ovariectomized mice, and this effect was reversed by the tamoxifen. In addition, immunohistochemical study indicated that ghrelin treatment reversed the reductions in c-Fos expression induced by ovariectomy. An estrogen antagonist tamoxifen also antagonized the effect of ghrelin on the c-Fos expression. Furthermore, the western blotting indicated that brain-derived neurotrophic factor (BDNF) in the hippocampus, but not phosphorylated cAMP response element-binding protein (pCREB)/CREB in the frontal cortex, were affected by ghrelin treatment. Ghrelin treatment significantly increased BrdU expression. Therefore, these findings suggest that ghrelin produces antidepressant-like effects in ovariectomized mice, and estrogen receptor may be involved in the antidepressant-like effects of the ghrelin.

## INTRODUCTION

Estrogen is well-known effects on modulating mood and emotion. For example, it has been reported that menopausal reduction in circulating estrogen levels are associated with an increase in mood disturbances in women [1, 2] and estrogen therapy sufficiently improves depressive symptoms [3]. In preclinical study, our group and other groups show that estrogen deficiency via ovariectomy causes an increase in depression-like behavior [4–6]. These changes in depression-like behavior seen in ovariectomized rats can be reversed by peripheral treatment with physiological doses of estradiol [4, 5]. These studies suggest that estrogen may be involved in estrogen deficient-induced depression-like behavior in ovariectomized rodents.

Ghrelin, a brain-gut peptide, is well known for its effects on hunger [7]. Several lines of studies show that females had significantly higher baseline concentrations of unacylated ghrelin than man [8, 9]. Moreover, treatment with estrogen for 8 h significantly increased the level of ghrelin expression, and ICI-182 780, an estrogen receptor antagonist, completely reversed this effect [10]. In our animal study, our findings show fasting also produce antidepressant like effect [11], and fasting also increases ghrelin levels in rodents and humans [12–15]. Another study also reported that ghrelin produces antidepressant-like effect in the normal mice [16]. Therefore, The present study was aimed to investigate the antidepressant-like effect of ghrelin in the estrogen deficient mice induced by ovariectomy.

As we described, the strong correlation between estrogen and ghrelin is no doubt. The antidepressant effect of estrogen has been proved to result in the up-regulation of brain-derived neurotrophic factor (BDNF) and decrease the 5-HT2A levels [17, 18]. Not only the levels of BDNF, evidences have proven that E2 increased cAMP response element- binding protein (CREB) expression, as well as CREB phosphorylation [19]. Furthermore, the effects of estrogen in hippocampal neurogenesis have also been reviewed [20, 21]. Interestingly, these mechanisms of estrogen-mediated anti-depression also reported in the antidepressant role of ghrelin. Thence, we have the hypothesis that estrogen-mediated antidepressant-like effect of the ghrelin. In this study, the mechanism of estrogen-mediated anti-depression of ghrelin in ovariectomized mice was investigated.

The expression of c-Fos immunohistochemistry is widely used to label neuronal activation in the brain [22, 23]. Acute antidepressant treatment increase c-Fos expression [24]. Furthermore, peripheral administration of ghrelin was also affected c-Fos expression in the mice brain [25]. Therefore, c-Fos is used to examine the antidepressant-like action of ghrelin in ovariectomized mice brain. In addition, decreased cell proliferation has also been reported in response to both acute and chronic stress paradigms [26] and antidepressant treatment increases neurogenesis in adult rat hippocampus [27]. Bromodeoxyuridine (BrdU), a thymide analog incorporated during the S-phase of cell division, was used as a marker of actively proliferating cells. In the recent study it have been reported that ghrelin increase BrdU expression in the mice hippocampus [28, 29]. Therefore, in this study, BrdU is used to test the effect of ghrelin on cell proliferation in ovariectomized mice.

CREB (cAMP response-element binding protein)-BDNF (brain derived neurotrophic factor) pathway is a well known pathway in depression. A reduced BDNF mRNA level was also reported in both the prefrontal cortex and hippocampus [30] and BDNF protein immunoreactivity was elevated in postmortem tissue from antidepressant treated patients [31]. On the other hand, decreased hippocampal CREB was found in temporal cortex or hippocampus in depressed patients studied at autopsy [32] and antidepressant treatment also increase CREB [33, 34]. CREB, in turn, induced several targets gene, including BDNF [35]. Recent studies have shown that ghrelin stimulated CREB through the activation of cAMP [36]. However, the role of CREB-BDNF pathway in antidepressant-like effect of the ghrelin was seldom reported in the ovariectomized mice.

In the present study, we aimed to investigate the antidepressant-like effect of ghrelin in the ovariectomized mice. Additionally, the effect of ghrelin on the c-Fos expression and BrdU were examined in the brain. Furthermore, the effect of the ghrelin on CREB-BDNF pathway is also investigated.

## RESULTS

### Effect of ghrelin on behavioral tests

### Open field test

**Figure 1:**
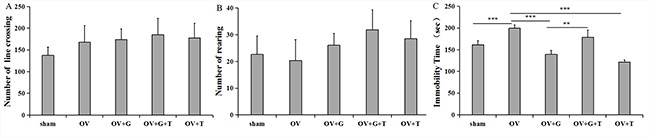
Ghrelin alleviate depression behavior (**A**) Locomotor behavior (number of line crosses) in the open field. (**B**) Rearing behavior (frequency) in the open field. Group conditions are indicated by abbreviations, and doses by numbers. (**C**) Group conditions are indicated by abbreviations, and doses by numbers. Sham: sham treatment; OV: ovariectomy; G: Ghrelin (1 mg/kg); T: tamoxifen (15 mg/kg). Values are mean ± S.E.M, *n* = 5–8, Symbols represent significant *post hoc* comparisons: Tukey's HSD, **P < 0.01 and ***P < 0.001.

We performed behavioral tests to determine the effect of ghrelin and tamoxifen on depression. To assess possible interferences on locomotor activity, the open field test was performed before the forced swim test. Figure 1A and 1B show that ghrelin has no effect on locomotor activity or rearing in the open field test (locomotor activity: *F_(4, 37)_* = 0.3659, *p = 0.83*; rearing:*F_(4, 37)_* = 0.4632, *p = 0.76*). These finding indicate that general changes in activity were not responsible for differences in immobility time induced by ghrelin in the forced swim test.

### Forced swim test

Immobility time were measured 30mins after the administration of ghrelin. Figure 1C shows that there were substantial differences in immobility time across treatment groups (*F_(4, 28)_* = 7.838, *p = 0.0003*). Ovariectomy increased immobility time compared to the sham treatment group (*p = 0.001*, Tukey's HSD), and ghrelin (1 mg/kg) significantly decreased ovariectomy-induced increase in immobility time (*p* < 0.001, Tukey's HSD). Furthermore, tamoxifen (15 mg/kg) reversed the effect of ghrelin on immobility time (*p = 0.008*, Tukey's HSD). Interestingly, this reversal normalized immobility compared to the sham control group. Tamoxifen alone decreased immobility time compare to the ovariectomized group (*p* < 0.001).

### Effect of ghrelin on c-Fos expression

**Figure 2:**
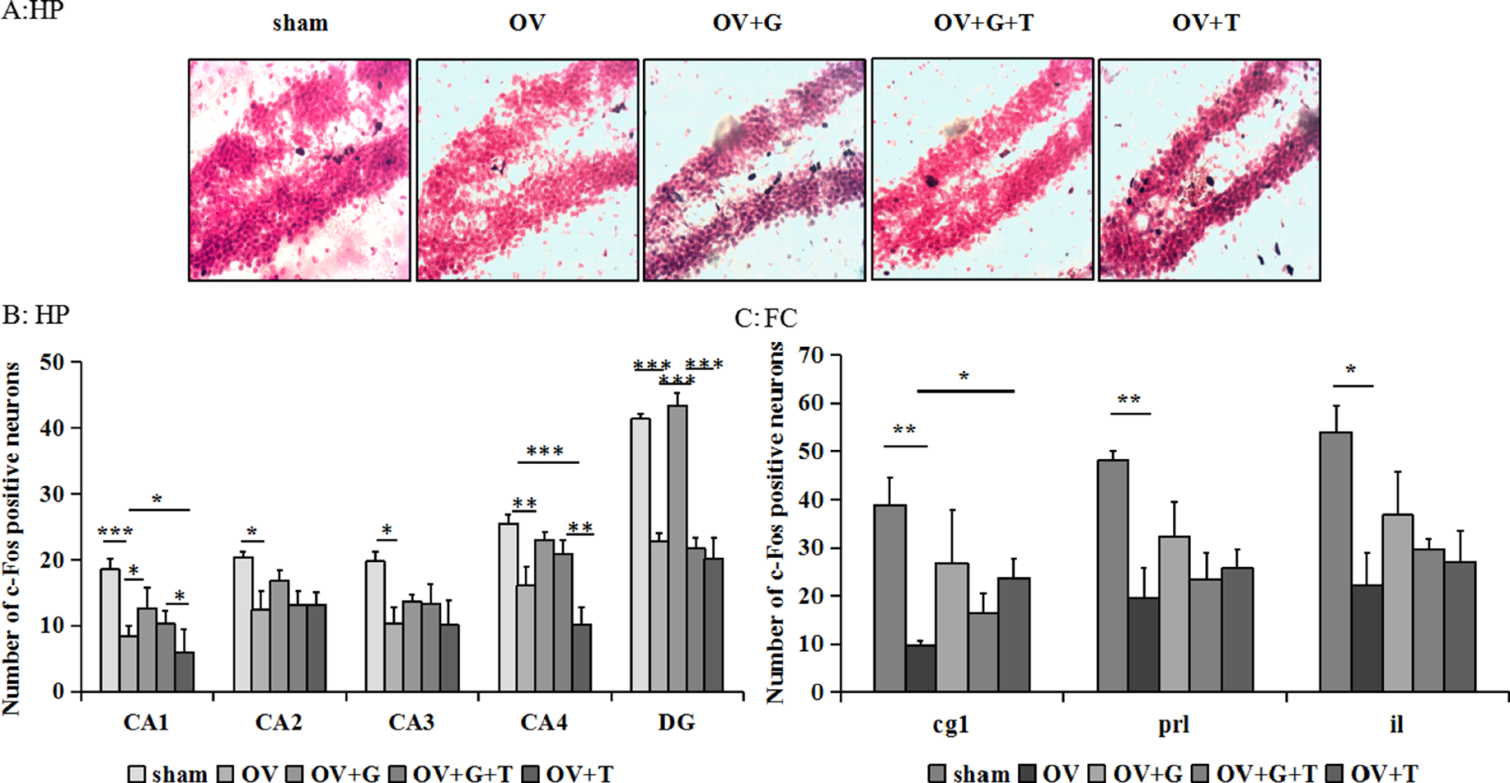
Ghrelin increases the c-Fos-positive cells in hippocampus (**A**) Representative sections through the dentate gyrus (inset: greater magnification near the tip of the dentate), showing c-Fos stained cells (dense brown nuclear staining) and counterstaining with neutral red. (**B**) The number of c-Fos positive cells in the dentate gyrus of the hippocampus. (**C**) The number of c-Fos positive cells in the subregions of the frontal cortex: Cg1, IL, and PrL. Group conditions are indicated by the following letters and abbreviations. HP: hippocampus; FC: frontal cortex; Sham: sham treatment; OV: ovariectomy; G: Ghrelin (1 mg/kg); T: tamoxifen (15 mg/kg). Values are mean ± S.E.M, *n* = 3–11, Symbols represent significant *post hoc* comparisons: Tukey's HSD, *P < 0.05, **P < 0.01 and ***P < 0.001.

**Figure 3:**
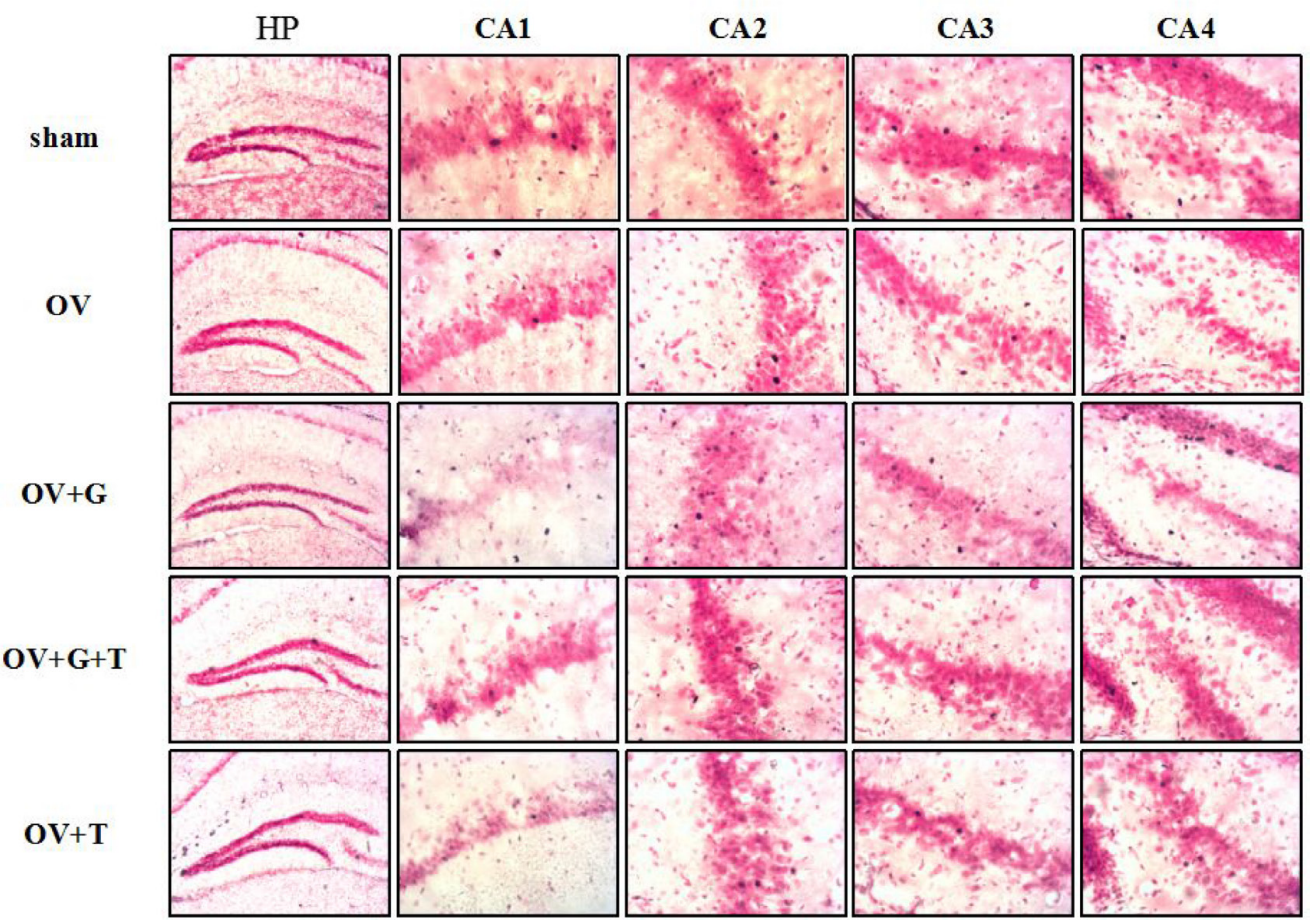
Ovariectomy decreases the c-Fos-positive cells in hippocampus Representative sections through the hippocampus (such as: CA1, CA2, CA3, CA4), showing c-Fos stained cells (dense brown nuclear staining) and counterstaining with neutral red. Group conditions are indicated by the following letters and abbreviations. HP: hippocampus; CA1, field CA1 of hippocampus; CA2, field CA2 of hippocampus; CA3, field CA3 of hippocampus; CA4, field CA4 of hippocampus; Sham: sham treatment; OV: ovariectomy; G: Ghrelin (1 mg/kg); T: tamoxifen (15 mg/kg). Values are mean ± S.E.M, *n* = 5–11, Symbols represent significant *post hoc* comparisons: Tukey's HSD, *P < 0.05, **P < 0.01 and ***P < 0.001.

Representative examples of c-Fos stained hippocampal sections are shown in Figure 2A and Figure 3, while Figure 2B and 2C represent the results of the cell counts, showing that there were significant differences across treatment groups in hippocampal and frontocortical subregions. In frontocortical subregions, ovariectomy reduced c-fos expression in the cingulate cortex (Cg1) (*F_(4, 20)_* = 4.081, *p = 0.018*; *p = 0.0086* vs. ovariectomized alone; Tukey's HSD), infralimbic cortex (IL) (*F_(4, 20)_* = 3.161, *p = 0.032*; *p = 0.022* vs. ovariectomized alone; Tukey's HSD) and prelimbic cortex (PrL) (*F_(4, 20)_* = 3.468, *p = 0.043*; *p = 0.0051* vs. ovariectomized alone; Tukey's HSD). Ghrelin and tamoxifen did not significantly alter the effects of ovariectomized in the Cg1, IL or PrL. Significant differences in c-Fos were observed in the dentate gyrus *(F*_(4,38)_ = 14.70, *p* < *0.001)*, CA1(*F*_(4,38)_ = 14.72, *p* < *0.001*), CA3(*F*_(4,38)_ = 3.977, *p = 0.0094*), CA4(*F*_(4,38)_ = 7.399, *p = 0.0002*) of the hippocampus. Ovariectomy produced a significant reduction in c-Fos expression only in the dentate gyrus (*p = 0.0002* vs. sham, Tukey's HSD), CA1 (*p* < 0.001 vs. sham, Tukey's HSD), CA3 (*p = 0.013* vs. sham, Tukey's HSD), CA4 (*p = 0.0036* vs. sham, Tukey's HSD) of the hippocampus. This effect was reversed by ghrelin in the dentate gyrus (*p = 0.0002* vs. ovariectomized alone, Tukey's HSD) and CA1 (*p = 0.014* vs. ovariectomized alone, Tukey's HSD) and then tamoxifen (*p = 0.0003*, vs. ovariectomized/ghrelin-treated mice, Tukey's HSD) prevented the effect of ghrelin on c-fos expression in the dentate gyrus. Although tamoxifen decreased c-Fos expression in the CA1 and CA4, there is no effect on the dentate gyrus of the hippocampus.

### Effect of ghrelin on BrdU expression

**Figure 4:**
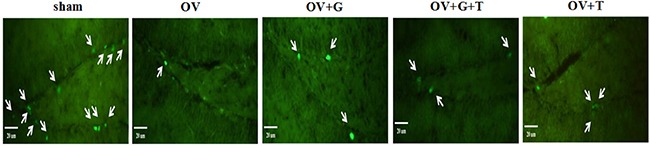
Ghrelin increased BrdU expression in the dendate gyrus of the hippocampus The arrows show BrdU stained cells. Group conditions are indicated by the following letters and abbreviations: sham (sham treatment), OV (ovariectomy), G (Ghrelin, 1 mg/kg) and T (tamoxifen, 15 mg/kg).

To determine whether ghrelin has a direct role in the proliferation of mice hippocampal NSCs, mice were adiministered BrdU at the beginning. As shown in Figure 4, ovariectomy decreased BrdU expression. Ghrelin treatment increased BrdU expression and this effect was antagonized by tamoxifen.

### Effect of ghrelin on the pCREB/CREB ratio

**Figure 5:**
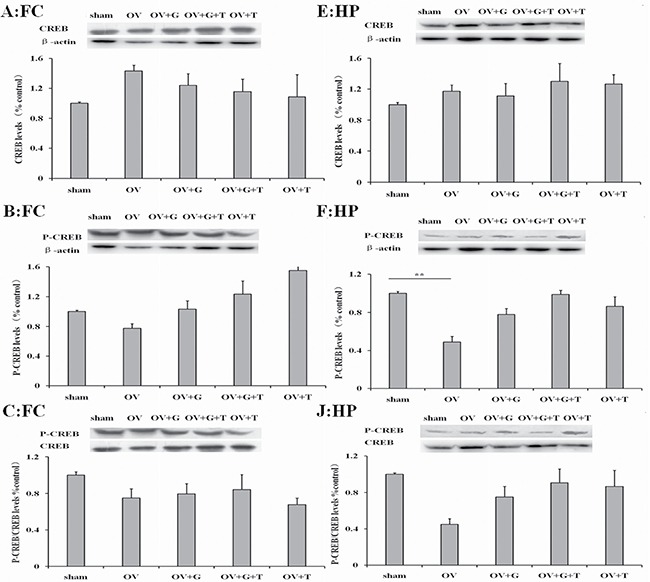
Ghrelin possible involve CREB signaling pathway Figures represent the CREB, pCREB and pCREB/CREB ratios in the frontal cortex (**A**–**C**) and hippocampus (**E**, **F** and **J**). Group conditions are indicated by abbreviations and doses by numbers. HP: hippocampus; FC: frontal cortex; Sham: sham treatment; OV: ovariectomy; G: Ghrelin (1 mg/kg); T: tamoxifen (15 mg/kg). Values are mean ± S.E.M, *n* = 4, Symbols represent significant *post hoc* comparisons: Tukey's HSD, **P < 0.01.

We next examine the protein of signal passageway whcih ghrelin may influence. Figure 5 shows western blotting results for pCREB (phosphorylated CREB and total CREB in the frontal cortex (Figure 5A–5C) and hippocampus (Figure 5E, 5F and 5J). Representative western blots are shown above and averages for each treatment group are shown. Significant differences in pCREB levels were observed in the hippocampus (treatment: *F_(4,19)_* = 3.186, *p = 0.044*), but not in the frontal cortex (*F_(4,19)_* = 0.4326, *p = 0.78*). Ovariectomy reduced the pCREB (*p = 0.0053*, Tukey's HSD) in the hippocampus. This effect was not reversed by ghrelin. There are no significant differences in total CREB or pCREB/CREB ratios in the hippocampus for each treatment group. Tamoxifen alone tendency to increase pCREB and pCREB/CREB ratios in the hippocampus.

### Effect of ghrelin on BDNF protein level

**Figure 6:**
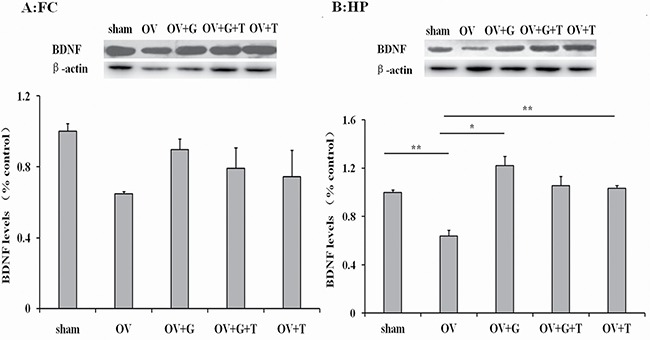
Ghrelin possible involve BDNF signaling pathway Figures represent the BDNF levels (normalized to β-actin) in the frontal cortex (**A**) and hippocampus (**B**). Group conditions are indicated by abbreviations and doses by numbers. HP: hippocampus; FC: frontal cortex; Sham: sham treatment; OV: ovariectomy; G: ghrelin (1 mg/kg); T: tamoxifen (15 mg/kg). Values are mean ± S.E.M, *n* = 4, Symbols represent significant *post hoc* comparisons: Tukey's HSD, *P < 0.05 and **P < 0.01.

Substantial differences in BDNF levels (normalized to β-actin levels) were observed across treatment groups in both the frontal cortex (*F_(4,19)_* = 0.5364, *p = 0.71*) and hippocampus (*F_(4,19)_* = 4.721, *p = 0.011*), as is shown in Figure 6. Ovariectomy reduced BDNF levels in the hippocampus (*p = 0,0085* Tukey's HSD). Ghrelin normalized BDNF levels in the hippocampus (*p = 0.012* vs. ovariectomized alone, Tukey's HSD) and this effect was not reversed by tamoxifen. Tamoxifen alone increases BDNF levels in the hippocampus (*p = 0.0068* Tukey's HSD).

## DISCUSSION

In this study, ghrelin shows antidepressant effects based on its ability to decrease immobility time in the forced swim test in ovariectomized mice. Our findings are consistent with previous results indicating that ghrelin produces antidepressant-like effect in the mice forced swimming test and *Ghsr*^−/−^ mice showed increased social avoidance after chronic social defeat stress [16]. Therefore, these findings suggest that the ghrelin may represent a novel potential treatment for mood disorders.

To consider potential mechanisms, c-Fos expression in the hippocampus and frontal cortex was examined. Consistent with behavior data, c-Fos expression was decreased by ovariectomy and reversed by ghrelin in the dentate gyrus of the hippocampus, and tamoxifen antagonized the effect of the ghrelin. These results support a role of the ghrelin exerts an antidepressant-like effect through the estrogen receptor after ovariectomy-induced estrogen deficiency mice, and hippocampus may be the one of the action site.

In this study, ovariectomy-induced decreasing effect of the BrdU was increased by single ghrelin treatment, and this effect was blocked by tamoxifen. BrdU is proliferation of the neuron, *as* proliferative *markers of* adult neurogenesis. Several reports have shown that the estrogen deficiency, induced by ovariectomy or postpartum estrogen withdrawal, regulates adult hippocampal neurogenesis [37–39]. Furthermore, systemic administration of ghrelin stimulates proliferation of newly generating cells in the hippocampus of adult mice [28]. In addition, immunoneutralization of ghrelin by using anti-ghrelin antiserum reduces proliferation of hippocampal progenitor cells in the subgranular zone [28]. Taken together, neurogenesis is involved in estrogen-mediated antidepressant-like effect of the ghrelin in ovariectomized mice.

The mechanisms that might underlie these effects of ghrelin are unknown. Several of those were explored in the present study. In the western blotting studies, ghrelin increases the BDNF in the hippocampus, which is reduced by ovariectomy. These results are consistent with previous reports that antidepressant treatments increase BDNF in the hippocampus [33, 40]. In addition, it has been reported that levels of BDNF are reportedly reduced in the hippocampus in depressed patients studied at autopsy [31]. These effects are similar to our findings here. However, tamoxifen did not reverse the effect of the ghrelin in the ovariectomized mice. This also needs further investigation.

In addition, the effect of the estrogen receptor antagonist tamoxifen on antidepressant-like effect of the ghrelin was examined. Tamoxifen at least partially reversed the effects of the ghrelin on the reductions in c-Fos induced by ovariectomy in the dentate gyrus. The results parallel the effects of ovariectomy and these drugs in the forced swim test, where tamoxifen antagonized the immobility-decreasing effects of ghrelin on elevations in immobility induced by ovariectomy. After ovariectomization, ghrelin expression was found to be decreased in a time dependent manner [41]. Lutter reported that ghrelin may have antidepressant effects in models of behavioral despair and chronic stress [16]. These findings suggested that ghrelin's antidepressant actions also may be direct and/or indirect activation of orexin-containing neurons, which are required for the antidepressant-like effect of calorie restriction [16]. In our previous study, fasting increased plasma estrogen levels and 17β-estradiol produces additive effect with fasting on the estrogen level [11]. It is well known that fasting could increase ghrelin level. Therefore, it is reasonable to assume that estrogen at least in part mediated antidepressant-like effect of the ghrelin.

Concerning tamoxifen alone treatment group, tamoxifen reduced depressive-like behavior in the forced swim test. The results are consistent with previous report [42]. However, it has been reported that tamoxifen by itself produced no changes in immobility time in the forced swim test [43]. Tamoxifen alone tendency to increase pCREB, pCREB/CREB and significantly increased BDNF levels in the hippocampus in the ovariectomized mice. These results may be consistent with our behavior study.

In this study, our findings suggest that ghrelin produces antidepressant-like effects in ovariectomized mice, and estrogen receptor may be associated with the antidepressant-like effects of the ghrelin. In some vitro experiments, it can be observed that ghrelin was secreted to medium by follicles collected and stimulated estradiol secretion *in vitro* ovarian follicles culture [44, 45]. In addition, ghrelin was shown to regulate the proliferation and apoptosis of porcine ovary cells [46]. Therefore, ghrelin may be a new potential antidepressant treatment. Ghrelin, an endogenous antidepressant, must have its benefits, but the potential side effects of ghrelin are needed to be concerned. As we mentioned above ghrelin is known for its effects on hunger. It has been observed that patients with chronic respiratory failure gained weight after ghrelin administration in a few clinical studies [47]. Besides, ghrelin may play a different role under physiologic or pathologic conditions [48]. Ghrelin can produce a significant antidepressant-like effect on chronic social defeat mice. Olfactory bulbectomized mice and chronic unpredictable mild stress rodents [16, 48, 49]. However, in most normal animals without stress, ghrelin may elicit anxiety-like behaviors [50–52]. Therefore, clinical correlations, along with a safety profile of ghrelin are warranted before it is put to use in patients.

## MATERIALS AND METHODS

### Animals

Female ICR mice (9 weeks old) were obtained from Jilin University (Changchun, China). Mice were housed in plastic cages (25.5 × 15 × 14 cm), and maintained in standard laboratory conditions, the temperature maintained at 23 ± 1°C, on a 12h light/dark cycle (lights on at 7 AM, lights off at 7 PM), with free access to food and water. The surgical procedure for ovariectomy followed our previous report by [53], in which the ovary was resected bilaterally in body weight of 35g mice. Briefly, each female mouse was anesthetized with 10% choloral hydrate [54]. Following a 0.5–1 cm-incision made with a small pair of scissors and forceps, the ovary was pulled through the opening in the musculature. A ligature was placed around each ovary and fallopian tube before the ovaries, and periovarian fat, were resected bilaterally. Sham-operated animals underwent the same procedure as the ovariectomized mice but without resection of the ovaries.

The study was conducted in accordance with the Guide for the Care and Use of Laboratory Animals published by National Institutes of Health and with the recommendations and approval of the Ethics Committee on Animal Experiments of the Jilin University. All efforts were made to minimize suffering.

### Drugs

Ghrelin was purchased from the tocris Bioscience (Bristol, UK). Tamoxifen was purchased from Sigma Aldrich Co. (St. Louis, MO, USA). All drugs dissolved in saline. All drugs administered intraperitonealy.

### Experimental design

In the behavioral portion of the study, sham ovariectomized animals served as control subjects for the effects of ovariectomy. There were 5 ovariectomized/drug treatment groups, including sham, ovariectomized/saline, ovariectomized/ghrelin, ovariectomized/ghrelin/tamoxifen, ovariectomized/tamoxifen group. In the behavior study, two groups of subjects were treated with ghrelin (1 mg/kg i.p.). ovariectomized/ghrelin/tamoxifen group of mice was treated with ghrelin (1 mg/kg i.p.) and tamoxifen (15 mg/kg i.p.). Tamoxifen was administered 30 min after ghrelin administration in the ghrelin/tamoxifen group. Doses of the ghrelin and tamoxifen were used according to the previous report [16, 46].

For the western blotting portion of the study all subjects that were tested behaviorally, and tissue collected from the prefrontal cortex and hippocampus. All mice were weighed daily throughout the period of drug treatments.

### Forced swim test

The forced swim test was performed 30 min after the ghrelin treatment in the same manner as our previous report [6]. It was carried out in a cylindrical container (11 cm in diameter, 25 cm high) filled with water to the height of 20 cm, and maintained at 25 ± 1°C. After the test, animals were dried with a towel and kept warm before returning them to home cages. Immobility times were manually recorded during the 6 min swim test from captured video of the test. The duration of immobility during the last 4 min of the trial was measured.

### C-Fos immunohistochemistry

Different from behavior test, new mice were used in c-Fos immunohistochemistry. Immunohistochemistry for c-Fos was performed as described in our former study [55]. Briefly, all mice were first deeply anesthetized with chloral hydrate (400 mg/kg, i.p.), and then decapitated. Brain perfusion was performed with ice-cold PBS, followed by 4% paraformaldehyde in PBS and post-fixed with 30% sucrose. Brains were cut in 30-μm sections on a vibratome. After rinsing sections in PBS, the sections were incubated with primary antibody (#sc-52;1:1000 dilution in PBS containing 0.3% Triton X-100, 0.05% sodium azide, and 2% normal goat serum) for 72 h at 4°C. The sections were then rinsed and incubated with a secondary antibody (biotinylated goat anti-rabbit IgG (Vector Laboratories); 1:400 dilution in PBS with 0.3% Triton X-100) for 75 min at room temperature. After rinsing in PBS, they were transferred into PBS containing 0.4% avidin-biotinylated horseradish peroxidase complex (Vector Laboratories) for another 75 min. Immunoreactivity was visualized using a glucose oxidase-diaminobenzidine-nickel method. The sections were counterstained with neutral red, graded alcohol series, cleared in xylene and cover slipped. The positive cells were counted under ×200 magnification from prefrontal cortex to hippocampus. Separate counting was done in prefrontal subregions, including Cg1, IL and PrL, according the atlas of Franklin and Paxinos [56]. Separate counts in the hippocampus were performed in the dentate gyrus, CA1, CA2, CA3, and CA4 region. C-Fos positive neurons were identified by their characteristic dense brown nuclear staining using light microscopy and captured with a connected Nikon digital camera (EcLipse 50i Microsope Nikon). These counts were averaged for 5–10 sections in each region for each animal.

### BrdU immuno-staining

Mice were adiministered 150 mg/kg (i.p.) BrdU on day 1 and 50 mg/kg on day 2. Mice were anesthetized with chloralhydrate (400 mg/kg, i.p.) and perfused transcardially with 4% paraformaldehyde 1h after the last- injection of BrdU. Coronal dorsal hippocampal slices (20μm) were cut using vibratome (Leica, CM1860). The dorsal dendate gyrus were each harvested on the basis of the coordinates of the mouse brain (Franklin and Paxinos). The free-floating sections were treated with deoxyri-bonucleic acid by incubation for 2 h in 50% formamide / 2 × standard saline citrate at 65°C followed by a 2 × SSC rinse. Sections were incubated for 30 min in 2 N HCl, PBS and for 15 min in 0.1 M boric acid, pH 8.5. After incubation in 3% normal goat serum in PBST (10 mM PBS containing 0.3% Triton X-100), and then incubated with mouse anti-BrdU antibody(1:800; Abcam; ab6326) overnight at 4°C. The sections were incubated in *Alexa* Fluor^®^ 488 goat anti–rat IgG (1:500; molecular probes; A11006) for 2 h. Immunoreactivities were visualized using an avidin-biotin horseradish peroxidase complex (Vector Laboratories, Inc, Burlingame, CA, USA). BrdU-positive cells in dorsal hippocampus were counted using a fluorescence microscope (Olympus, Tokyo) to analyze the sections at a magnification of ×400.

### Western blot of CREB, pCREB and BDNF

Immediately after forced swimming, frontal cortex and hippocampus were collected and the protein extracted using standard procedures. Tissue lysates were assessed for levels of BDNF, CREB and pCREB by Western blot. After separation using 10% SDS-PAGE gels, proteins were transferred to polyvinylidene difluoride fmembranes by electroblotting. BDNF, CREB, pCREB and β-actin were immunostained by initial incubation with the following primary antibodies: BDNF (1:1000, rabbit polyclonal; santacruzbio, sc546), pCREB-ser133 (1:1000, rabbit polyclonal; Cell signaling, CST9197S), CREB (1:1000, rabbit monoclonal; Cell signaling (CST9191S) and β-actin(1:2000, Mouse monoclonal; transgen Biotech; #HC201). The membranes were incubated with the respective peroxidase labeled secondary antibodies (anti rabbit: 1:400; proteintech, SA00001-2). The bands were visualized on Tanon-5200 Chemiluminescent Imaging System (Tanon Science & Technology Co., Ltd). Specific band densities were quantified using image J software and the ratio between the intensity of pCREB/CREB and BDNF/ β-actin were calculated [11].

### Statistical analyses

All values are presented as the mean ± S.E.M. The significance of the data was analyzed using one-way analysis of variance. One-way analysis of variance were use in behavior, immunohistochemistry and western blot study. When significant differences were obtained, *post hoc* comparisons were performed using *Tukey’s* honestly significant difference test (*Tukey’s HSD*), comparing treatment groups and sham-treated animals, and between ovariectomized/ghrelin-treated subjects and ovariectomized/ghrelin/tamoxifen-treated subjects. *P* values less than 0.05 were considered significant.
